# Outcomes from deep brain stimulation targeting subthalamic nucleus and caudal zona incerta for Parkinson’s disease

**DOI:** 10.1038/s41531-019-0089-1

**Published:** 2019-08-21

**Authors:** Abteen Mostofi, Julian M. Evans, Lucy Partington-Smith, Kenny Yu, Cliff Chen, Monty A. Silverdale

**Affiliations:** 1 Manchester Centre for Clinical Neurosciences, Salford Royal Hospital, Stott Lane, Salford, Greater Manchester M6 8HD UK; 2 Manchester Academic Health Science Centre, University of Manchester, Manchester, UK; 3 Present Address: Neurosciences Research Centre, St George’s, University of London, Cranmer Terrace, London, SW17 0RE UK

**Keywords:** Parkinson's disease, Parkinson's disease, Neurological manifestations, Parkinson's disease, Brain

## Abstract

Both subthalamic nucleus (STN) and caudal zona incerta (cZI) have been implicated as the optimal locus for deep brain stimulation (DBS) in Parkinson’s disease (PD). We present a retrospective clinico-anatomical analysis of outcomes from DBS targeting both STN and cZI. Forty patients underwent bilateral DBS using an image-verified implantable guide tube/stylette technique. Contacts on the same quadripolar lead were placed in both STN and cZI. After pulse generator programming, contacts yielding the best clinical effect were selected for chronic stimulation. OFF-medication unified PD rating scale (UPDRS) part III scores pre-operatively and ON-stimulation at 1–2 year follow up were compared. Active contacts at follow-up were anatomically localised from peri-operative imaging. Overall, mean UPDRS part III score improvement was 55 ± 9% (95% confidence interval), with improvement in subscores for rigidity (59 ± 13%), bradykinesia (58 ± 13%), tremor (71 ± 24%) and axial features (36 ± 19%). Active contacts were distributed in the following locations: (1) within posterior/dorsal STN (50%); (2) dorsal to STN (24%); (3) in cZI (21%); and (4) lateral to STN (5%). When contacts were grouped by location, no significant differences between groups were seen in baseline or post-operative improvement in contralateral UPDRS part III subscores. We conclude that when both STN and cZI are targeted, active contacts are distributed most commonly within and immediately dorsal to STN. In a subgroup of cases, cZI contacts were selected for chronic stimulation in preference. Dual targeting of STN and cZI is feasible and may provide extra benefit compared with conventional STN DBS is some patients.

## Introduction

Deep brain stimulation (DBS) of the subthalamic nucleus (STN) is effective in improving the motor complications of Parkinson’s disease (PD) and is in widespread clinical use.^1–5^ The sensorimotor subregion of STN is located in its dorsolateral aspect and is commonly targeted in DBS for PD.^6–8^ The zona incerta is a region of loosely arranged cell groups lying ventral to the thalamus and dorsal to STN, in continuity with the thalamic reticular nucleus. Its posterior part extends over the dorsum of STN to form a discrete region—the caudal zona incerta (cZI), part of the region known as the ‘posterior subthalamic area’—which lies immediately posteromedial to STN.^9^ Its connections are diffuse but include the basal ganglia, motor areas of cerebral cortex and thalamus and the cerebellar nuclei.^10–12^ Stimulation of cZI has been recently shown to be more effective than best medical management in motor improvement, with a particularly marked benefit in tremor.^13^ Furthermore, a study comparing motor outcomes from DBS targeting cZI versus dorsolateral STN in separate cohorts of patients suggested that stimulation of cZI yielded significantly greater benefit than that of dorsolateral STN.^14^

We have performed DBS targeting the subthalamic region bilaterally as the routine surgical treatment of idiopathic PD in our centre since 2011. Given the reported discrepancies in the optimal stimulation locus, electrode trajectories were planned to place the contacts of a quadripolar lead within both dorsolateral STN and cZI. Here, we retrospectively report our clinical outcomes with this approach. We perform a pragmatic observational analysis of the anatomical location of the electrode contacts yielding the best clinical effect and therefore selected for chronic stimulation (the ‘active’ contacts) post-operatively in order to shed light on which subthalamic locus is the more effective target. We examine the interaction between active contact location and underlying motor characteristics of the study patients.

## Results

### Efficacy of DBS targeting STN and cZI

The primary outcome measure of total UPDRS part III score in the entire study population post-operatively ON-stimulation/OFF-medication was significantly improved compared with the OFF-medication pre-operative baseline (mean score from 55.2 to 24.7, *p* < 0.001), with a mean relative improvement of 55 ± 9% (95% confidence interval). There were significant improvements in subscores for rigidity (59 ± 13%), bradykinesia (58 ± 13%), tremor (71 ± 24%) and axial symptoms (36 ± 19%). Scores in UPDRS parts I (evaluation of mentation, behaviour and mood), II (self-reporting of activities of daily living) and IV (complications of therapy) also saw highly significant improvements following DBS surgery of 26 ± 14%, 24 ± 11% and 41 ± 16%, respectively (summarised in Table 1). This clinical benefit was accompanied by a 32 ± 8% mean reduction in the levodopa equivalent daily dose (LEDD), with LEDD increasing post-operatively in only a single patient.

**Table 1:** UPDRS outcome data and levodopa equivalent daily dose (LEDD) for all 40 patients

	Baseline score, mean	Post-DBS score, mean	Mean percent improvement (95%C.I.)
UPDRS-III	55.2	24.7	55% (46–65)
Rigidity	10.8	4.4	59% (46–72)
Bradykinesia	21.0	8.7	58% (45–72)
Tremor	9.7	2.8	71% (47–95)
Axial	10.2	6.5	36% (17–55)
UPDRS-I	13.4	10.0	26% (11–40)
UPDRS-II	19.8	15.1	24% (12–35)
UPDRS-IV	8.8	5.2	41% (25–57)
LEDD/mg	1233	825	32% (24–40)

Mean baseline pre-operative OFF-medication and post-operative ON-stimulation/OFF-medication UPDRS part III total score and subscores for rigidity, bradykinesia, tremor and axial features are shown. Mean baseline pre-operative and post-operative scores for UPDRS parts I, II and IV and LEDD are also presented. All comparisons between baseline and post-DBS scores were highly significant (*p* < 0.001; paired *t*-test)

### Localisation of active contacts

**Fig. 1:**
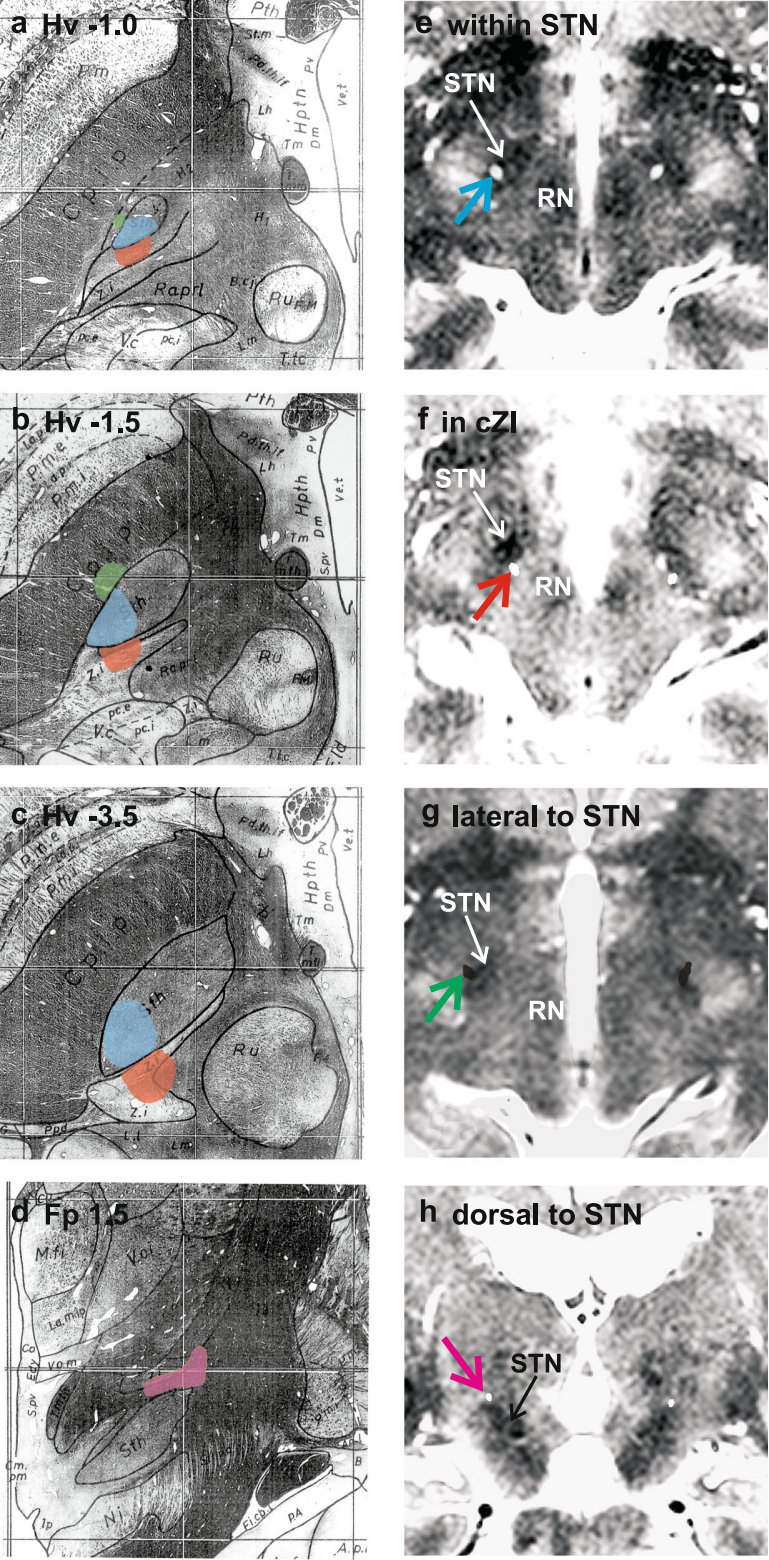
Anatomical locations of the active contacts were divided into four groups, the site and extent of each indicated by coloured shading in representative labelled example sections (**a**–**c** axial, **d** coronal) from the stereotactic atlas of Schaltenbrand and Wahren^21^: within STN (blue); dorsal to STN (purple); posteromedial to STN, in cZI (red); and lateral to STN (green). **e**–**h** Typical examples of images used to localise electrodes is presented for each active contact location. The intra-operative study is fused with the planning MRI scan in the plane of the active contact centroid. The stylette artefacts (coloured arrows; colours as in **a**–**d**) from the intra-operative imaging (CT study in **e**, **f** and **h**; MRI study in **g**) are thus visible on the anatomical planning scans and allow contact localisation. **e**–**g** show axial and **h** coronal slices. STN and red nucleus (RN) are labelled

**Fig. 2:**
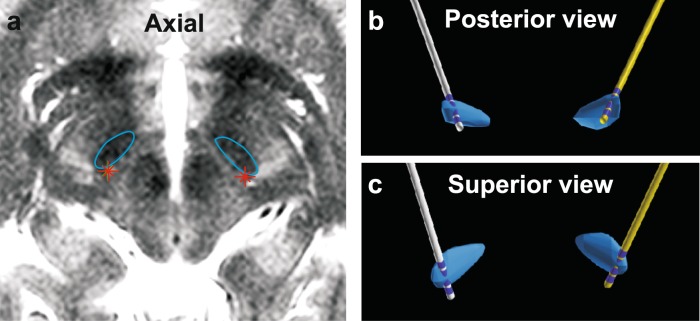
Typical planned electrode trajectories for bilateral subthalamic DBS. **a** Axial T2-weighted MRI with STNs marked in blue outline. Red asterisks represent the cZI target loci posteromedial to STN. Trajectories illustrated relative to STN (rendered blue volume) viewed posteriorly **b** and superiorly **c** on digital three-dimensional reconstructions from the planning software. The four contacts on each quadripolar Medtronic 3389 lead are visible. The distal contact 0 lies at the cZI target locus, more proximal contacts 1 and 2 within STN and the most proximal contact 3 just dorsal to STN

We were able to divide the 76 reconstructed active contact loci into four groups based on the anatomical location of the contact centroid: (1) dorsal to STN (18 electrodes; 24%); (2) within STN, typically posteriorly and dorsally (38 electrodes; 50%); (3) posteromedial to STN, corresponding to the location of cZI or ‘posterior subthalamic area’ (16 electrodes; 21%); and (4) immediately lateral to STN (4 electrodes; 5%). The site and extent of these regions are summarised in standard atlas representations in Fig. 1a–d with specific examples from the source imaging illustrated in Fig. 1e–h. As would be expected, active contacts in the region dorsal to STN were more proximal on the quadripolar lead (median contact 3) than those within STN (median contact 2) and those in cZI tended to be even more distal (median contact 1; *p* < 0.001 for trend, Kruskal–Wallis test), reflecting the planned incorporation of these regions in a typical electrode placement (Fig. 2).

Mean *X* (lateral), *Y* (anterior) and *Z* (superior) coordinates of the centroid of the active contact for the four anatomical locations, relative to each individual’s mid-commissural point in the AC–PC plane, in millimetres, were: dorsal to STN (13.2, −1.2, 0.2); within STN (12.9, −2.9, −2.1); cZI (12.1, −4.5, −2.2); and lateral to STN (14.0, −1.2, −1.7). Note that the *X*-coordinates for both right- and left-sided electrodes are expressed as positive values for comparability.

### Relation of active contact location to clinical features

We assessed whether active contact location influenced the motor improvement seen post-operatively. UPDRS part III rigidity, bradykinesia and tremor subscores were determined for limbs contralateral to each of the active contacts, as was the sum of the three subscores (the ‘total’ score). Scores for active contacts lateral to STN were excluded from statistical analysis due to the paucity of data points. For the remaining three groups (dorsal to STN, within STN and in cZI), significant improvements attributable to stimulation were seen in all motor subscores within all groups (Table 2). No significant inter-group differences in any of the subscores were seen in the percentage individual improvement seen ON-stimulation. There was also no difference in post-operative LEDD reduction between the groups (*p* > 0.05, Kruskal–Wallis test).

**Table 2:** Median and interquartile range (IQR) of baseline pre-operative OFF-medication and post-operative ON-stimulation/OFF-medication UPDRS part III subscores for contralateral (C/L) rigidity, bradykinesia and tremor, and their sum total (total UPDRS-III), for the three groups of active contacts located dorsal to STN, within STN and in cZI

	Baseline score, median (IQR)	Post-DBS score, median (IQR)	Individual percent improvement, median (IQR)	*p*-value
**C/L rigidity**				
Dorsal to STN	4 (1–6)	1 (0–3)	40% (25–100)	0.001
Within STN	5 (3–6)	2 (1–3)	60% (33–83)	<0.001
In cZI	5 (3–6)	2 (0–2)	75% (38–100)	<0.001
Inter-group *p*-value	0.308		0.529	
**C/L bradykinesia**				
Dorsal to STN	9 (4–14)	3 (1–3)	68% (21–79)	0.001
Within STN	11 (7–15)	3 (2–6)	70% (33–82)	<0.001
In cZI	9 (6–15)	3 (2–5)	62% (34–80)	0.001
Inter-group *p*-value	0.450		0.886	
**C/L tremor**				
Dorsal to STN	5 (1–9)	1 (0–2)	75% (8–93)	0.001
Within STN	3 (0–4)	0 (0–1)	100% (50–100)	<0.001
In cZI	0 (0–7)	0 (0–2)	71% (50–91)	0.031
Inter-group *p*-value	0.066		0.287	
**C/L total UPDRS-III**				
Dorsal to STN (*n* = 18)	17 (12–24)	5 (3–7)	65% (50–74)	<0.001
Within STN (*n* = 38)	19 (13–24)	6 (4–10)	69% (37–78)	<0.001
In cZI (*n* = 16)	18 (11–22)	6 (3–9)	72% (51–75)	<0.001
Inter-group *p*-value	0.810		0.916	

Also shown are medians and IQRs of the individual percentage improvement in the scores seen post-operatively. Intra-group pre- and post-operative absolute scores are compared and *p*-values are shown in the rightmost column (two-tailed Wilcoxon signed-rank test). Pre-operative baseline scores and post-operative individual percentage improvement in scores are compared between groups (inter-group *p*-values shown; Kruskal–Wallis test) with no significant inter-group differences

Given the lack of a clear relationship with motor improvement, we asked whether the baseline pre-operative motor phenotype influenced the location of the active contact selected during pulse generator programming. Pre-operative OFF-medication contralateral UPDRS part III rigidity, bradykinesia and tremor subscores and their sum (the ‘total’ score) were compared across the three groups. Again, no significant inter-group differences were seen (Kruskal–Wallis test; see Table 2).

Next, we determined if active contact location was associated with any differences in stimulation parameters. This was based on the theoretical consideration that if one of these locations is the optimal stimulation target, then greater stimulation intensity may be expected at the other more distant loci to achieve the equivalent clinical effect seen. Stimulation frequency and pulse width were invariant across all electrodes in our patients at 130 Hz and 60 μs, respectively. Median stimulating current was lowest for the active contacts within STN (2.34 mA) versus those dorsal to STN (2.71 mA) and in cZI (2.97 mA), though the trend was not statistically significant (*p* = 0.087, Kruskal–Wallis test).

We noted that amongst the 16 leads with active contacts in cZI, six did not have proximal contacts with centroids within STN. In these six leads, proximal contacts were immediately adjacent to the superomedial border of STN. For these leads, therefore, it cannot be known if stimulation within STN, as defined in this study, would be superior to that of cZI. As a consequence, we repeated the above analyses whilst excluding these six leads from the cZI group. With this condition, we again found no significant inter-group differences in pre-operative motor scores, post-operative motor improvement or stimulation parameters between the groups of contacts (*p* > 0.05, Kruskal–Wallis test). We also compared, within the cZI group, the pre-operative motor scores, post-operative motor improvement and stimulation parameters between the subgroup of leads with proximal contacts within STN (*n* = 10) and that without (*n* = 6). No significant differences were seen between these two subgroups of cZI active contacts (*p* > 0.05, Wilcoxon rank-sum test).

### Safety and accuracy of the technique

In the 40 patients included in this study, one patient (2.5%) required a second procedure to re-implant the guide tubes that were inaccurately placed due to intra-operative displacement of the stereotactic frame. Two patients (5%) underwent skin erosion at the pectoral IPG site requiring IPG removal. One patient (2.5%) experienced pectoral skin erosion managed surgically and with prolonged intravenous antibiotics without IPG removal. On the intra-operative imaging studies, no intracranial haematomas, pneumocephalus or significant brain shift were demonstrated.

Mean scalar error in electrode placement was 1.3 ± 0.1 mm (95% confidence interval) with no significant difference between left- and right-sided electrodes (*p* = 0.483, two-sample *t*-test). Fifty seven percent of electrodes were placed within one electrode diameter (1.27 mm) and 99% within two diameters in the axial plane of the planned cZI target locus.

## Discussion

In this ‘real-world’ retrospective observational study, we report our outcomes from bilateral subthalamic DBS for PD in which we have intentionally targeted both STN and cZI on a single trajectory. This targeting strategy was motivated by the fact that while there is a large body of high-quality evidence supporting STN as a target for DBS for PD,^1–5^ others have achieved good results with cZI stimulation^13–15^ and one group has reported superior efficacy with cZI compared with STN.^14^ On a pragmatic level, the mean improvement in the overall OFF-medication UPDRS part III score of 55% from baseline in the whole study population compares well against published data from bilateral STN DBS.^4,5,16,17^ This shows that our targeting strategy produces outcomes no less favourable than when STN is targeted conventionally. This improvement is reflected in all four cardinal motor domains in keeping with previous accounts of STN DBS. Significant improvements are also seen in motor fluctuations as assessed by the part IV (complications of therapy) score, and in non-motor assessments reflected in the part I (mentation, behaviour and mood) and part II (activities of daily living) scores of the UPDRS, replication of which in the literature has been variable.^2,4,5,16,17^

We are only the second group to report a large series of DBS operations using an image-verified implantable guide tube/stylette method described previously.^18–20^ The technique’s benefits include the ability to localize stylette location readily on intra-operative magnetic resonance imaging (MRI) or computed tomography (CT) without electrode artefact, as exploited in this study, and the potential to revise hardware without the need to repeat the stereotactic process. We have demonstrated that this method can be performed safely, accurately and effectively in a second centre and is therefore scalable.

Our major objective in this study was to review the locations of active contacts selected during pulse generator programming for chronic stimulation in order to shed light on which out of STN and cZI is the more frequently stimulated and therefore more effective target. In this regard, we performed a pragmatic retrospective clinico-anatomical analysis utilising routinely collected clinical data. At the time of follow up when contact selection and programming is expected to have been optimised, we determined the location of the active electrode contacts selected for chronic stimulation to identify which loci were being stimulated. We demonstrate that with our targeting strategy, the most frequent locus for active contacts is within the dorsal and posterior part of STN, as seen in half of our electrode implantations. This area is in keeping with the sensorimotor part of STN previously described and targeted.^7,8^ This was followed by the region immediately dorsal to STN and ventral to the thalamus in approximately one quarter of leads. In this area resides the rostral zona incerta located in between fields H2 and H1 of Forel in which pallido- and cerebello-thalamic projection fibres are found.^10,21^ Some previous studies examining the relationship between active contact location and therapeutic effect have implicated this region as the most effective locus, and activation of pallidofugal fibres has been suggested as a putative mechanism.^22–26^

These two sites—STN and its dorsal border area—together account for almost three quarters of the implanted leads. Therefore, targeting the STN and placing contacts within the nucleus and over its dorsal border—as would normally be performed in conventional STN DBS—appears to be more important than targeting cZI in the majority of cases. This contradicts a previous finding demonstrating the superiority of cZI stimulation over that of STN.^14^ However, in approximately one in five of our implanted leads, the active contact was located in cZI. In the majority of cases where cZI was selected for chronic stimulation, the cZI contact was selected in preference to proximal contacts in STN. This implies that stimulation of cZI conferred more benefit than that of the other regions during pulse generator programming in this subgroup of implantations. We therefore propose that targeting cZI in addition to STN, as we report in this study, may provide a more effective stimulation locus for a minority of implanted leads that may benefit a sizeable fraction of patients undergoing DBS therapy for PD.

We sought to determine if the location of active contacts was related to clinical features seen in the study population. We demonstrate no significant association between active contact location and contralateral motor improvement, neither in the total change in contralateral UPDRS part III score nor in the subscores for rigidity, akinesia and tremor. In other words, as long as the most effective contact is selected during programming for chronic stimulation, its specific location—be it dorsal to STN, within STN or in cZI—does not influence the derived motor benefit (although a recent study suggests non-motor improvement may be affected^27^). Furthermore, the pre-operative contralateral motor scores were not different between patients with active contacts in the three different areas, suggesting that pre-operative motor phenotype is not a significant determinant of the most effective stimulation locus. We are therefore unable to shed light on why a particular contact is chosen over the others in individual patients when across the population active contacts are located in these different regions. The choice of contact used may well be determined more by the side effect profile of stimulation rather than by the motor features of PD that are explored in this study, however we were unable to examine this further from the clinical records available. The therapeutic effect of cZI stimulation may lie in the extent of activation of fibre bundles within the activated field, namely the thalamic and lenticular fasciculi.^11^ These bundles cannot be resolved directly with conventional clinical imaging, which may account for some variability in contact placement relative to these structures and impact upon the relative efficacy of cZI versus STN stimulation in individual cases.

Localisation of the active contacts using the method described above is subject to potential limitations and sources of error, namely that are introduced by MR distortion and CT-MRI image co-registration. Co-registration accuracy for plain CT and MRI using the same software as this study has recently been reported as well within the sub-millimetre range.^28^ Our own preliminary data suggest that our method of using a CT angiogram may provide even further accuracy by allowing vasculature close to the region of interest to provide feature detail for improved co-registration. We infer that the implanted electrode will be in exactly the same location as the stylette. This relies on the demonstrated effectiveness of our precautions for preventing intra-operative brain shift. We have defined an active contact’s location based on its centroid. In reality, it is a cylinder of length 1.5 mm and diameter 1.27 mm and the volume of tissue it activates will be even larger than this. Therefore, the volume of tissue activated by a contact will be greater than the point locus representing its location and may include structures neighbouring that in which the locus resides. Electrical stimulation parameters were not significantly different between the three main groups, rendering it unlikely that the distribution of active contact locations can be accounted for fully by spread of current to a true single ‘best’ locus nearby. However, it must be acknowledged that the lack of statistical difference between groups could be attributed to the sample size in this study. Further investigation of DBS targeting different subthalamic structures in larger numbers of patients may yield further insights into the key determinants of target selection.

In conclusion, in this pragmatic observational study of DBS for PD, we demonstrate that our method of dual targeting of the STN and cZI is safe, accurate and effective, with outcomes that compare favourably to existing published data on STN DBS. The negative contact yielding the best clinical effect and chosen for chronic stimulation is located within the STN or dorsal to it in around three out of four cases. The cZI contact is chosen in preference to contacts within and dorsal to the STN in approximately one fifth of implantations. We therefore propose that targeting cZI in addition to STN on a single trajectory is practicable and provides an extra therapeutic option that may yield greater benefit than conventional STN DBS for some patients.

## Methods

### Patients

We present a detailed clinical and anatomical targeting analysis of our first 40 patients implanted with DBS electrodes for idiopathic PD as part of their routine clinical care. Patients were selected based on well-established criteria for DBS by a mutli-disciplinary team.^29^ Baseline characteristics of participants are summarised in Table 3. All patients gave written informed consent for the use of anonymised routine clinical data for audit and research. This study was approved by the Manchester Centre for Clinical Neurosciences, Salford Royal NHS Foundation Trust, Greater Manchester, United Kingdom.

**Table 3:** Baseline characteristics of the 40 patients in the study at the time of surgery

**Age/years (mean** **±** **SD)**	59 ± 9
**Sex**	
Male	28 (70%)
Female	12 (30%)
**Duration of disease/years (mean** **±** **SD)**	9 ± 3
**Baseline LEDD/mg (mean** **±** **SD)**	1233 ± 608 mg
**Hoehn and Yahr stage (median, range)**	2 (1–5)
**Indication for DBS**	
Motor fluctuations and dyskinesia	33 (83%)
Disabling tremor	7 (17%)

*LEDD* levodopa equivalent daily dose,^30^ *SD* standard deviation

### Pre-operative planning and target definition

A planning MRI study of the brain acquiring high-resolution 1.5T T2-weighted (repetition time 4525 ms, echo time 140 ms) axial, coronal and sagittal, and volumetric gadolinium-enhanced T1-weighted images (repetition time 20 ms, echo time 4.6 ms) was obtained under general anaesthesia. Target loci in cZI were identified immediately medial to the medial border of the posterior third of STN at the level of the maximal diameter of the red nucleus in the axial plane parallel to the anterior commissure—posterior commissure (AC–PC) line. Trajectories with frontal and lateral entry points were planned for quadripolar leads (Model 3389, Medtronic, USA) using dedicated software (neuroinspire™, Renishaw, UK). Trajectories were drawn typically to position the distalmost contact 0 within the target cZI locus and the more proximal contacts 1 and 2 within the posterior third of STN (Fig. 2). Typical trajectory angles were 35–50° anterior in the sagittal plane and 15–30° lateral in the coronal plane in Cartesian space defined by the AC–PC plane, and skull entry points were anterior to the coronal suture.

The first ten patients underwent the planning MRI study on the day of surgery in a Leksell stereotactic frame (Co-ordinate Frame G, Elekta, Sweden). For subsequent patients it was performed without the frame days prior to surgery and was co-registered with a stereotactic CT angiogram (0.625 mm slice thickness) in the Leksell frame obtained on the day of surgery, using the Circle of Willis as a region of interest.

### Surgical technique and peri-operative imaging

Bilateral DBS leads were implanted under general anaesthesia based on the guide tube/stylette method previously described by others^20^ (neuroguide™ system, Renishaw, UK). A plastic guide tube cut 12 mm shorter than the planned trajectory length was inserted stereotactically towards the target over a rigid probe and bonded to the skull with acrylic bone cement. A plastic stylette was inserted through each guide tube along the planned trajectory and its location verified with intra-operative stereotactic imaging in the frame (volumetric gadolinium-enhanced T1-weighted MRI scan for the first ten patients, and volumetric CT scan for the remainder). If targeting accuracy on the intra-operative imaging study was deemed satisfactory, the stylettes were removed and DBS leads were passed down the guide tubes to exactly the same depth and connected via extension leads to a pulse generator (Activa PC or Kinetra, Medtronic, USA) subcutaneously implanted in the chest or abdominal wall.

### Stimulator programming

Around four weeks after surgery, participants were admitted for stimulator programming. Programming was performed using a standardised protocol. Patients were programmed in the OFF-medication state (after 12 h withdrawal of PD medication). The left-sided lead was tested initially using monopolar review of each contact separately from distal to proximal (contacts 0, 1, 2 and 3). Initial pulse width was 60 µs and frequency was 130 Hz. The stimulator amplitude was then gradually increased from 0 to 6 V checking for any beneficial effects and any side effects. All contacts were tested systematically, before deciding on the optimal contact. The optimal contact was chosen as the one that provided good symptom control and a large therapeutic window (no side effects up to 6 V, or side effects occurring at least 1.5 V higher than beneficial effects). If no optimal contact could be found after monopolar testing of all contacts, then bipolar and tripolar stimulation was attempted. Subsequently the right-sided lead was systematically tested in a similar systematic fashion.

### Determination of electrode active contact locations

For 38 patients (76 leads) the location of the implanted stylettes was able to be verified accurately. In the remaining two patients the intra-operative imaging allowed broad verification of implantation accuracy but was not of sufficient resolution to allow detailed reconstruction of stylette placement. In each patient, the intra-operative imaging study was co-registered with the pre-operative planning MRI study. The trajectories of the stylettes—and hence of the leads—were defined in Cartesian coordinates relative to the AC–PC plane and mid-commissural point as determined on each patient’s own planning MRI study (*X*, right lateral; *Y*, anterior; *Z*, superior). The active (negative) contact and stimulation parameters at the time of follow-up clinical assessment between one and two years post-operatively were obtained from the clinical notes. The anatomical location of each active contact centroid was retrospectively determined and visualised on a fused image of the planning MRI and intra-operative imaging study. This location was transposed onto the representative Schaltenbrand and Wahren atlas section^21^ for illustrative purposes using the axes of STN as a guide for the transposition as described by others previously.^14^ In order to evaluate the implantation technique used, any discrepancies between planned and actual trajectories were quantified in terms of Euclidean vector error in *X* and *Y* dimensions of the centre of the electrode shaft from the planned cZI target locus in the same axial (*Z*) plane. The magnitude of this vector error is the reported scalar error. Confidence limits for targeting error were derived using the *t*-distribution.

### Outcome measures

Movement disorder society unified PD rating scale (UPDRS) scores were assessed before surgery (baseline) and between one and two years post-operatively. All assessments were performed by a single investigator (L.P.-S.) who was blinded to the location of the active contact in post-operative patients. Median interval between surgery and assessment at follow-up was 16.5 months (range 11–27 months). The primary outcome measure was the total UPDRS part III (clinician-scored motor evaluation) score ON-stimulation/OFF-medication at follow-up versus the pre-operative OFF-medication score. Secondary outcome measures included post-operative OFF-medication change in UPDRS part III subscores for bradykinesia (items 3.4–3.8), rigidity (3.3), tremor (3.15–3.18) and axial symptoms (3.9–3.14), and post-operative change in UPDRS parts I (evaluation of mentation, behaviour and mood), II (self-evaluation of activities of daily living) and IV (complications of therapy). Mean baseline and post-operative scores were compared using a paired two-tailed *t*-test (*n* = 40) and 95% confidence intervals for the mean change derived from the *t*-distribution.

We investigated whether differences in active contact location impacted upon the motor improvement seen post-operatively. The electrodes were grouped based on the anatomical location of the centroid of each active contact. Within each group, baseline OFF-medication and post-operative ON-stimulation/OFF-medication UPDRS part III subscores for bradykinesia, rigidity and tremor pertaining to the contralateral limbs were compared, as was the sum of three subscores (the ‘total’ score; Wilcoxon signed-rank test). For each subscore, inter-group differences in the individual post-operative percentage changes from baseline and the pre-operative baseline scores were examined (Kruskal–Wallis test). The active contacts lateral to STN were excluded from statistical analysis due to paucity of observations.

### Reporting summary

Further information on experimental design is available in the Nature Research Reporting Summary linked to this article.

## Supplementary information

Reporting Summary

## Data Availability

The data that support the findings of this study are available from the corresponding author upon reasonable request.
